# Complete genome sequence of *Marinobacter shengliensis* D49 harboring *ectABC* genes for ectoine synthesis

**DOI:** 10.1128/MRA.00414-23

**Published:** 2023-09-21

**Authors:** Shohei Yasuda, Akihiko Terada

**Affiliations:** 1 Department of Applied Physics and Chemical Engineering, Tokyo University of Agriculture and Technology, Naka-Cho, Koganei, Tokyo, Japan; 2 Global Innovation Research Institute, Tokyo University of Agriculture and Technology, Harumi-Cho, Fuchu, Tokyo, Japan; Montana State University, Bozeman, Montana, USA

**Keywords:** *Marinobacter*, ectoine, resource recovery, isolation, hybrid assembly

## Abstract

A complete genome sequence of *Marinobacter shengliensis* D49 in the class *Gamma-proteobacteria* was isolated from activated sludge treating landfill leachate. The genome encodes the functional genes for the biosynthesis of ectoine (*ectABC*), a compatible solute for cosmetics. Deciphering the genome helps pave the way for ectoine production by the isolate.

## ANNOUNCEMENT

*Marinobacter shengliensis* D49 was isolated from an enriched biomass with a cell sorter (On-chip Biotechnologies Co., Ltd., Tokyo, Japan). The biomass was inoculated from an aeration tank in a landfill leachate treatment plant (Tokyo, Japan). The sorted biomass was incubated with DSMZ1180 medium (https://www.dsmz.de/microorganisms/medium/pdf/DSMZ_Medium1180.pdf) to imitate the salinity condition of the leachate with shaking at 130 rpm for 10 days at 30°C. Subsequently, the grown cells were harvested by centrifugation at 10,000 rpm for 5 min. The genomic DNA was extracted by a phenol-chloroform extraction technique (1). The RNA contaminants in the genomic DNA were depleted by RNaseA (TaKaRa Bio, Inc., Shiga, Japan). For long-read sequencing, the library was prepared using a 1D ligation sequencing kit (SQK-LSK-109; Oxford Nanopore Technologies Ltd., Oxford, UK) without a fragmentation procedure. MinION Mk1B using an R.10 flow cell (FLO-MIN110; Oxford Nanopore Technologies Ltd., Oxford, UK) was applied for the sequencing, and the sequence data were base-called by Guppy (ver. 5.0.11) (2), obtaining 358 K reads (3.62 Gbp). The sequence quality was confirmed by NanoPlot (ver. 1.40.0) (3), and the adaptor sequences, low-quality reads (<Q12), header (75 bp), and short reads (<10,000 bp) were removed with NanoFilt (ver. 2.8.0) (3), resulting in 35.1 K reads (989.5 M bp). Regarding a short-read sequencing procedure, the library preparation proceeded with the same DNA extract as the long-read sequencing according to the manufacturer’s protocol of an MGIEasy Universal DNA Library Prep Set (MGI Tech., Shenzhen, China). Subsequently, 150 bp paired-end sequencing was performed with DNBSEQ-G400RS (MGI Tech., Shenzhen, China) by a sequencing service (Genome-Lead Co., Ltd., Kagawa, Japan), obtaining 2.48 M reads (0.95 Gbp). The adapter and barcode sequences, low-quality reads (<Q30), one base pair at the tail end, reads shorter than 20 bp, and reads that have more than 10 numbers of N bases were removed using fastp (ver. 0.23.2) (4). Hybrid assembly processed with Unicycler (ver. 0.5.0) (5) using the long- and short-reads generated a circularized chromosome and two plasmids. The genome completeness (100%) was assessed by gVolante (ver. 2.0.0) with BUSCO v1 pipeline (6), and the taxonomy was classified as *M. shengliensis* using GTDB-Tk (ver. 2.0.1) (7) with a 97.5% average nucleotide identity (ANI) value. The amino acid sequence was obtained with DFAST (—aligner blastp) (ver. 1.2.17) (8), and the functional annotation was proceeded with using BlastKOALA (ver. 3.0) (Release 2023–04-01) (9). Default parameters were employed for all software unless otherwise specified. The genomic information is listed in Table 1. The genome of *M. shengliensis* consists of a chromosome and two plasmids with each length of 4,305,073 bp, 157,124 bp, and 13,198 bp, respectively.

**TABLE 1:** The genomic information of *M. shengliensis* D49.

Type	Name	GC%	Size (Mb)	Average covarage depth	rRNAs	tRNAs	Other RNA	CDSs
Chromosome	-	57.3	4,305,073	141	9	53	-	3,910
Plasmid	pMAD49a	53.3	157,124	73	-	-	-	165
Plasmid	pMAD49b	57.5	13,198	1,368	-	-	-	17

Based on the gene mapping to reference pathways of KEGG (Release 106.0) (10), *M. shengliensis* D49 harbors *ectABC* functional genes encoding ectoine (*ectABC*) biosynthesis (11), suggesting potential for producing ectoine using the isolate. Deciphering the complete genome of *M. shengliensis* D49 will lead to harnessing the isolate for ectoine production from landfill leachate.

## Data Availability

The complete genome sequence of *M. shengliensis* D49 has been deposited as three contigs in DDBJ/EMBL/GenBank under the accession numbers AP028062, AP028063, and AP028064. The BioSample accession number SAMD00599713, the BioProject accession number, PRJDB15760, and the Run accession numbers DRR463738 and DRR463739 were linked to the database.
